# The Potential Role of Protein Kinase R as a Regulator of Age-Related Neurodegeneration

**DOI:** 10.3389/fnagi.2021.638208

**Published:** 2021-04-28

**Authors:** Nicolás W. Martinez, Felipe E. Gómez, Soledad Matus

**Affiliations:** ^1^Fundación Ciencia & Vida, Santiago, Chile; ^2^Departamento de Ciencias Básicas, Facultad de Medicina y Ciencia, Universidad San Sebastián, Santiago, Chile; ^3^Biomedical Neuroscience Institute, Faculty of Medicine, University of Chile, Santiago, Chile; ^4^Center for Geroscience, Brain Health and Metabolism, Santiago, Chile

**Keywords:** double-stranded RNA-dependent protein kinase, integrated stress response, neurocognitive functions, Alzheimer’s disease, Parkinson’s disease, Huntington’s disease, aging

## Abstract

There is a growing evidence describing a decline in adaptive homeostasis in aging-related diseases affecting the central nervous system (CNS), many of which are characterized by the appearance of non-native protein aggregates. One signaling pathway that allows cell adaptation is the integrated stress response (ISR), which senses stress stimuli through four kinases. ISR activation promotes translational arrest through the phosphorylation of the eukaryotic translation initiation factor 2 alpha (eIF2α) and the induction of a gene expression program to restore cellular homeostasis. However, depending on the stimulus, ISR can also induce cell death. One of the ISR sensors is the double-stranded RNA-dependent protein kinase [protein kinase R (PKR)], initially described as a viral infection sensor, and now a growing evidence supports a role for PKR on CNS physiology. PKR has been largely involved in the Alzheimer’s disease (AD) pathological process. Here, we reviewed the antecedents supporting the role of PKR on the efficiency of synaptic transmission and cognition. Then, we review PKR’s contribution to AD and discuss the possible participation of PKR as a player in the neurodegenerative process involved in aging-related pathologies affecting the CNS.

## Introduction

Neurocognitive functions rely on neuronal cell adaptation mechanisms during the nervous system lifespan. Neurons respond continuously to changing cellular contexts to achieve these adaptive dynamics. An increasing evidence has shown a reduced capacity in adaptive homeostasis mechanisms in aging and aging-related neurodegenerative diseases. One of the signaling pathways that allow cell adaptation is the integrated stress response (ISR). The ISR comprises four kinases/sensors: double-stranded RNA-dependent protein kinase [protein kinase R (PKR)], heme-regulated inhibitor (HRI), the general control non-derepressible-2 (GCN2), and the PKR-like endoplasmic reticulum (ER) resident protein kinase (PERK). Depending on the stimulus, one of the ISR kinases becomes active and phosphorylates the eukaryotic translation initiation factor 2 (eIF2α) on Ser51 (Harding et al., 2003; Pakos-Zebrucka et al., 2016). This phosphorylation event not only leads to a decrease in global protein synthesis but also induces the expression of selected genes, including the one coding for the activating transcription factor 4 (ATF4) (Harding et al., 2003; Pakos-Zebrucka et al., 2016). Once translated, ATF4 translocates to the nucleus and promotes genetic programs involved in essential cellular processes/functions, including autophagy, redox homeostasis, and amino acid metabolism. However, under chronic overactivation, ISR can also induce apoptosis (Pakos-Zebrucka et al., 2016). Although ISR signaling events have been described in dividing cells, the consequences of ISR activation, specifically in neurons, remain poorly explored in physiological conditions. However, there are strong pieces of evidence that suggest a role for ISR in central nervous system (CNS) pathophysiology. For instance, there is significant information about the role of PERK in neurodegeneration. PERK is a sensor that can be activated directly by mis- or unfolded proteins at the ER (Wang et al., 2018). Several neurodegenerative diseases are characterized by the presence of aggregated unfolded or misfolded proteins, including Huntington’s disease (HD), Parkinson’s disease (PD), and AD, and the contribution of PERK has been largely discussed in other articles (Bell et al., 2016; Hughes and Mallucci, 2019). This review is focused on the kinase PKR and the consequences of its activation in the CNS. PKR is a stress sensor first identified as a kinase responding against viral infection (Meurs et al., 1990). Today, PKR is considered a significant regulator of central cellular processes, including mRNA translation, transcriptional control, apoptosis regulation, growth regulation, and cell proliferation (He et al., 2013; Zhou et al., 2014; Shinohara et al., 2015; Gal-Ben-Ari et al., 2018). In the CNS, PKR controls protein synthesis and regulates synaptic and cognitive function. PKR activation has also been involved in neurological conditions, including AD (Peel and Bredesen, 2003; Couturier et al., 2010; Dumurgier et al., 2013; Hugon et al., 2017) (reviewed in Hugon et al., 2017), PD (Bando et al., 2005), and HD (Peel et al., 2001; Bando et al., 2005), suggesting a role of PKR in pathologies associated to aging affecting the CNS.

Here, we reviewed the antecedents supporting PKR and the consequences of its activation as a key player in physiological conditions and its contribution in the neurodegenerative process involved in aging-related pathologies affecting the CNS.

## PKR-eIF2α Role on CNS Physiology

**FIGURE 1:**
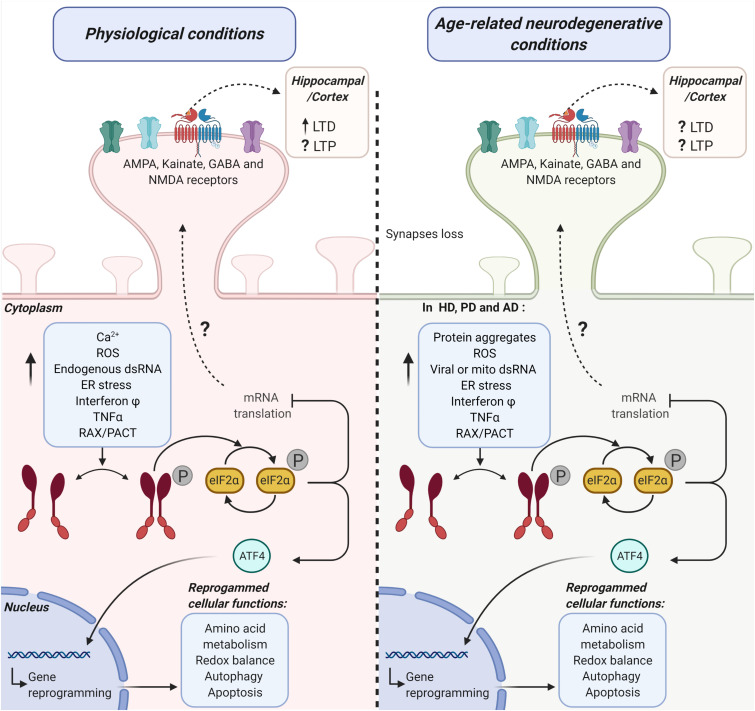
Possible mechanistic links between the activation of the PKR-eIF2α branch of the ISR and hippocampal/cortical plasticity under physiological and age-related neurodegenerative conditions. The diagram represents the potential role of the double-stranded RNA protein kinase [protein kinase R (PKR)] in synaptic transmission efficiency under physiological conditions (left side) and age-related neurodegenerative conditions (right side). PKR-activating stimuli induce a significant increase in the phosphorylation (P) of the alpha subunit eukaryotic initiation factor 2 (eIF2α) in a dsRNA-dependent manner or via its endogenous activator RAX/PACT. This pathway activation promotes the inhibition of mRNA translation and ATF4 dependent gene reprogramming of several cellular functions. Altogether, PKR-eIF2α ISR branch activation may lead to cell adaptation or cell death. PKR-eIF2α ISR branch loss of function (LOF) under physiological conditions (left side) significantly modifies cortical and hippocampal receptor-mediated synaptic transmission. Consequently, long-term potentiation (LTP) and long-term depression (LTD) are also affected. Under age-related neurodegenerative conditions (right side), common stimuli could participate in the activation of PKR-eIF2α ISR branch activation in Huntington’s diseases (HD), Parkinson’s disease (PD), and Alzheimer’s disease (AD). Under this activation, ATF4 translocates to the nucleus and mediates degenerative reprogrammation. On the other hand, the eIF2α phosphorylation decreases mRNA translation. This decrease in translation induces modifications in the hippocampal and cortex LTP and LTD through an unknown mechanism.

Protein kinase R is conserved in vertebrates, and it has not been found in plants, fungi, protists, and invertebrates (Taniuchi et al., 2016). PKR was initially described to be activated by viral double-stranded RNAs (dsRNA) (Hovanessian, 2007). Once the stimulus is detected, the activation of PKR is mediated by homodimerization and trans-autophosphorylation at threonine 446 and threonine 451 (Romano et al., 1998). Activated PKR induces the phosphorylation of eIF2α (p-eIF2α), leading to general protein synthesis inhibition (Romano et al., 1998). In parallel, PKR activation can also promote p-eIF2α-dependent translation of messenger RNAs (mRNAs) containing specific 5′ untranslated region (UTR) regulatory regions (Chesnokova et al., 2017). One of the proteins synthesized after ISR activation is ATF4, which leads to apoptosis in several cell types (Wek et al., 2006; Hussain and Ramaiah, 2007; Lee et al., 2007). It has been recently reported that PKR activation is negatively regulated by its interaction with sphingosine kinase 1 (SPHK1), which reduces apoptosis execution on cell lines (Qiao et al., 2021). It has been reported that PKR can also be activated in the absence of virus (dsRNA) by endogenous cellular stresses such as oxidative stress, intracellular calcium increase, or ER stress (Romano et al., 1998; Zhu et al., 2011; He et al., 2013). Additionally, interferon-gamma (IFNγ), tumor necrosis factor α (TNFα), heparin, platelet-derived growth factor, and inosine–uracil mismatches have also been described as PKR activators (Deb et al., 2001; Page et al., 2006; Anderson et al., 2011; McAllister et al., 2012; Husain et al., 2015; Reimer et al., 2018). Thus, the PKR-eIF2α branch may potentially be activated in response to a diversity of inductors depending on a particular cellular context. Moreover, a specific PKR activation mechanism has been described, in the absence of dsRNA, through the PKR associated protein activator (PACT) (in humans) or its murine homolog RAX. Thus, PACT/RAX has been proposed as physiological activators of PKR (Patel and Sen, 1998; Ito et al., 1999). A recent detailed study of PKR-activating dsRNAs found that most of the PKR-interacting RNA repertoires are mitochondrial RNAs (mtRNAs) that can form intermolecular dsRNAs (Youssef et al., 2015; Kim et al., 2018). mtRNAs interaction with PKR was found to induce this kinase activation, eIF2α phosphorylation, and subsequent control cell translation (Youssef et al., 2015; Kim et al., 2018). This promiscuity of PKR as a sensor of endogenous and exogenous stimuli suggests that PKR could be a negative regulator of protein synthesis in several cellular physiological and pathological contexts (see Figure 1).

As mentioned earlier, ISR activation can lead to cell adaptation or apoptosis, which has been extensively reviewed elsewhere (Pakos-Zebrucka et al., 2016). This paradoxical role of the ISR is supported by signaling assays on proliferating cells with spatially homogeneous dynamics. An ISR modulating stimulus is applied to a whole cell, and survival readouts are studied on these assays. In neuronal models, it has been established that the activation of the PKR eIF2 a branch also mediates apoptosis (Vaughn et al., 2014). However, it is worth considering that neuronal cells show a significantly higher degree of complexity to support their local cytoarchitecture. This particularity of neurons diversifies the potential roles of homeostasis regulation on neuronal functions. For example, it has been established that local protein synthesis, a process directly regulated by PKR, is necessary not only to maintain local axonal phenotype (merotrophism) and axonal integrity but also to execute axonal degeneration in an apoptotic independent manner (Alvarez et al., 2000; Whitmore et al., 2003; Hillefors et al., 2007; Pazyra-Murphy et al., 2009). Thus, local protein synthesis regulates neuronal integrity. Consequently, homeostasis regulation mechanisms may be essential not only for neuronal survival but also for local integrity maintenance in neurons. However, the participation of PKR in local integrity maintenance in neurons remains unknown.

The role of PKR-eIF2α in the CNS has been studied using genetic strategies. The mouse in which part of the PKR coding gene has been deleted (PKR-KO) or the knock-in mouse bearing an eIF2α allele with the S51A mutation (eIF2αS/A) does not show qualitative changes on CNS overall morphology and histological patterns of axonal or synaptic markers (Costa-Mattioli et al., 2007; Zhu et al., 2011) compared to wild-type (WT) mice. Specifically, PKR-KO mouse brain shows no gross abnormalities on Nissl staining and presynaptic or postsynaptic structures, visualized by synaptic markers such as the vesicular glutamate transporter 1 (VGLUT1), the postsynaptic density protein 95 (PSD95), and the glutamic acid decarboxylase 67 (GAD67), a GABAergic terminal marker (Zhu et al., 2011). Additionally, eIF2αS/A knock-in mouse has no detectable differences in the hippocampus overall morphology, based on axonal or presynaptic marker stains (Small et al., 2000; Costa-Mattioli et al., 2007). In spite of this general preservation of CNS tissue under PKR-eIF2α ISR branch loss of function (LOF), a growing body of evidence shows that PKR participates in neuronal essential functions, survival, and integrity.

### PKR in Neuronal Function

A major functional expression of synaptic plasticity is the efficiency of synaptic transmission. In turn, an efficient synaptic transmission supports neurocognitive functions. The relationship between synaptic plasticity and neurocognitive functions has been extensively reviewed before (Martinez et al., 2018). Importantly, synapses loss and dendritic atrophy are observed in the aging human brain and correlate with neurocognitive dysfunctions progression (Uylings, 2000). Consequently, altered synaptic transmission is also a common characteristic of age-related neurodegenerative diseases (Kumar and Foster, 2007). The widest used model for modifiability of synaptic transmission is the hippocampal field activity measurement, based on the synaptic plasticity role on cognitive memory mechanisms. There, brief high-frequency neuronal firings induce an increase in the efficacy of neuronal synapses that sustains for hours, known as long-term potentiation (LTP) (Connor and Wang, 2016; Peters et al., 2018). This physiological adaptive mechanism requires postsynaptic Ca^2+^ significant entry, activation of metabotropic glutamate receptors, and the generation of diffusible intercellular messengers (Landfield et al., 1986; Barnes et al., 1992; Rosenzweig et al., 1997; Connor and Wang, 2016; Peters et al., 2018). On the other hand, repeated low-frequency firing, which results in a modest rise in Ca^2+^, induces long-term depression (LTD), provoking a significant, sustained decrease in the efficacy of neuronal synapses. Healthy aging and age-related neurodegeneration are associated with a decrease in synaptic plasticity, leading to impaired synaptic transmission (Rosenzweig et al., 1997). Consequently, reduced ability to enhance synaptic transmission through LTP or an LTD imbalance over LTP would trigger a decrease in synaptic transmission in aged animals (Landfield et al., 1986; Barnes et al., 1992; Rosenzweig et al., 1997; Temido-Ferreira et al., 2019). LTP and LTD are the major mechanistic insights that have been proposed to correlate with performance on mouse models of cognitive memory, which has been already reviewed (Landfield et al., 1986; Barnes et al., 1992; Rosenzweig et al., 1997; Connor and Wang, 2016; Figlioli et al., 2019).

An accumulating evidence suggests that the PKR-eIF2α signaling pathway negatively regulates hippocampal synaptic transmission efficiency in non-pathological conditions. Earlier studies on hippocampal slices showed that a significant decrease in eIF2α phosphorylation levels correlates with a lower threshold for long-lasting LTP (LTP that requires protein synthesis) (Takei et al., 2001; Costa-Mattioli et al., 2005). This result suggests that the eIF2α pool phosphorylation state modulates the establishment of a sustained increase in the efficacy of neuronal synapses. Furthermore, the eIF2αS/A mouse, which shows reduced eIF2α phosphorylation levels (by ∼50%) relative to WT mice, does not display any difference in basal transmission compared to WT mice (Costa-Mattioli et al., 2007). Under these significantly reduced eIF2α phosphorylation levels, Schaffer collateral/commissural fibers’ stimulation with a short-lasting inducing LTP protocol (which does not require protein synthesis) (Kandel, 2001; Kelleher et al., 2004) elicits the expected result on WT hippocampal slices. Notably, the same protocol elicits a long-lasting LTP on hippocampal slices from eIF2αS/A mice littermates, which is sensitive to protein synthesis inhibitors (Costa-Mattioli et al., 2005, 2007). These results strongly suggest that eIF2α phosphorylation negatively regulates the transition from short- to long-lasting LTP at the hippocampus. Notably, previous reports have shown that eIF2α phosphorylation is necessary for LTD promotion at hippocampal slices (Di Prisco et al., 2014). Taken together, these results suggest that eIF2α phosphorylation could simultaneously modulate both LTP and LTD at the hippocampus. Consequently, ISR sensor kinases may potentially play a role in transmission efficiency by modulating the eIF2α phosphorylation state.

Acute pharmacological inhibition of PKR induces aberrant activity on the neocortex of free-moving adult mice, studied by electroencephalography (EEG), without changes in ongoing behavior (Zhu et al., 2011). These results suggest that eIF2α-mediated regulation of synaptic efficiency could be downstream of PKR signaling. Coincidently, authors have determined that PKR genetic deficiency in mice leads to aberrant hyperactivity of neuronal networks by reducing GABA-mediated inhibitory synaptic transmission (Zhu et al., 2011). In this model, PKR LOF led to a significantly higher number of spikes reaching a higher excitatory postsynaptic potential (ceiling), presumably because PKR-mediated inhibition was impaired (Zhu et al., 2011). Thus, the authors have proposed that the physiological role of PKR over transmission efficiency is to maintain a relatively low level of excitability by enhancing GABAergic synaptic transmission with a lack of change in postsynaptic receptor-related mechanisms.

As mentioned earlier, eIF2α phosphorylation is necessary to induce LTD (Di Prisco et al., 2014). Notably, PKR activation is sufficient to induce sustained LTD mediated by the phosphorylation of eIF2α (Di Prisco et al., 2014). LTD induction by PKR activation has been studied in a transgenic mouse in which a drug can activate PKR in certain neurons in the CNS. Specifically, the mouse expresses a transgene encoding a drug-dependent conditional PKR that dimerizes and activates at hippocampal CA1 neurons. The pharmacological induction of PKR activation led to eIF2α phosphorylation and sustained LTD selectively on neurons expressing the transgene (Di Prisco et al., 2014). Together, these data support the idea that PKR and eIF2α are major regulators of transmission efficiency. Thus, the regulation mediated by PKR-eIF2α also participates in CNS physiology at a functional level, beyond neuronal survival and integrity. Concomitantly, loss of function of eIF2α and PKR also induces significant changes over neurocognitive functions.

Since the modulation of eIF2α phosphorylation impacts transmission efficiency, it is plausible to expect that it also affects the performance of mice when paradigms evaluating cognitive memory are applied. In fact, it has been shown that pharmacological inhibition of eIF2α dephosphorylation mediates memory consolidation of drug-paired stimuli (Huang et al., 2016). In a model in which basolateral amygdala (BLA)-dependent cocaine addiction is established, rats are exposed to freely choose between saline paired side or cocaine paired side of a chamber. The difference in the time spent on the cocaine-paired side versus the saline-paired side is calculated as the cocaine place preference. Authors have found that eIF2α phosphorylation and ATF4 levels diminish in the BLA after a re-exposure to a previously cocaine-paired context (Huang et al., 2016). Notably, local injection in the BLA of a selective inhibitor of eIF2α dephosphorylation after this memory retrieval protocol disrupts drug-paired stimulus-induced craving (Huang et al., 2016). Moreover, eIF2α also plays a role in spatial learning and spatial memory when studied by the Morris water maze paradigm (Costa-Mattioli et al., 2007). In this test, mice are trained during consecutive days to swim, find a platform, and memorize its location in a pool. Then, the time required for the mice to find the hidden platform (“escape latencies”) and the time spent on the platform’s quadrant is measured (Morris et al., 1982). Using this setup, authors have found that eIF2αS/A knock-in mice reached the platform significantly faster than their WT littermates (Costa-Mattioli et al., 2007). In addition, eIF2αS/A mice had a significantly greater preference for the platform quadrant (Costa-Mattioli et al., 2007). These results strongly suggest that eIF2α partial LOF enhances hippocampal-dependent spatial learning and spatial memory. Furthermore, the eIF2α’s role over memory function has also been explored in several experimental paradigms of protein synthesis-dependent long-lasting memory such as auditory and contextual fear conditioning, conditioned taste aversion (CTA), and latent inhibition (LI) of CTA (Rosenblum et al., 1993; Bourtchouladze et al., 1998; Schafe et al., 1998) in eIF2αS/A mice. Remarkably, partial loss of eIF2α function (eIF2αS/A) induces a significant improvement on evocative parameters at all those above experimental long-lasting memory paradigms (Costa-Mattioli et al., 2007). Thus, eIF2α phosphorylation state modulation regulates long-term memory function beyond its effect on the transmission efficiency discussed before.

Similarly, acute pharmacological targeting of PKR using the inhibitor C16 has been evaluated in a taste learning paradigm. Local stereotaxic injection into the insular cortex or intraperitoneal (i.p.) injection of C16 before applying an aversive taste stimulus results in enhanced cortical-dependent novel taste learning and CTA in rats (Ariffin et al., 2010; Stern et al., 2013). This effect of acute inhibition has been proposed as indicative of PKR involvement in cognitive processing. To evaluate this, authors had used acute pharmacological inhibition of PKR before contextual and auditory fear conditioning and studied the incorporation of neurons to functional circuits induced by learning through the expression of a specific marker gene, immediate-early gene (Egr-1) (Hall et al., 2000; Frankland et al., 2004; Zhu et al., 2011). On this experimental setup, both contextual and auditory long-term fear memories were enhanced under PKR pharmacological inhibition in mice (Zhu et al., 2011). Concomitantly, in the same group of experiments, long-lasting memory in PKR-KO mice correlated with Egr-1 levels at hippocampal CA1 neurons. Authors have proposed that PKR LOF not only improves long-lasting memory but also participates in the recruitment of CA1 neurons into the cognitive encoding process (Zhu et al., 2011). Furthermore, the PKR-KO mouse also shows significantly improved hippocampus-dependent spatial memory assayed by the Morris water maze test. Even more, the PKR-KO mouse also shows improved auditory and contextual long-term fear memories compared to WT littermates when tested through Pavlovian fear conditioning (Zhu et al., 2011). Thus, genetic deletion of PKR strengthens long-term spatial memory.

### PKR on Glial Cells

Several authors have reported the presence of the components of the PKR-eIF2α branch on another type of cells present in the CNS, the glial cells (Auch et al., 2004; Ong et al., 2005; Alirezaei et al., 2007; Vantelon et al., 2007; Flores-Mendez et al., 2013). However, the role of the PKR-eIF2α branch on glial functions under physiological conditions remains mostly unexplored. In this context, emerging reports suggest that eIF2α activation controls protein a synthesis rates on glial cells. Specifically, protein synthesis rates increase in astrocytes correlate with Eif2 a phosphorylation, in response to lactic acid (Vantelon et al., 2007). It has also been reported that eIF2α activation mediates protein synthesis rate changes in response to glutamate neurotransmitter on Bergmann glial cells (Flores-Mendez et al., 2013). In turn, PKR mediates an increase in nitric oxide (NO) production on *in vitro* human astrocytes in response to dsRNA by inducing inducible nitric oxide synthase (iNOS) expression (Auch et al., 2004). Altogether, these reports suggest that the PKR-eIF2α branch may have a relevant role in CNS functions regulated by glial cells. Despite this, the antecedents available describing the role of the components of the PKR-eIF2α branch on glial biology have been performed in the context of pathological inflammation, which we will review in the next section.

**FIGURE 2:**
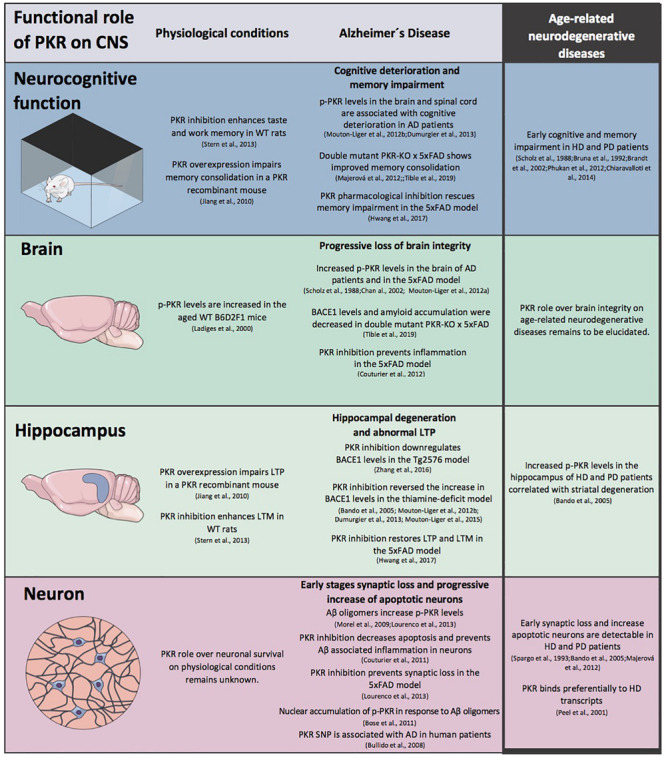
The role of protein kinase R (PKR) in CNS physiology and pathophysiology. A schematic view of the modulation exerts by PKR in CNS morpho-functional integrity at different levels under physiological conditions and in Alzheimer’s (AD) disease. The information available for age-related neurodegenerative diseases is included.

Altogether, an accumulated evidence suggests that the PKR-eIF2α signaling pathway participates in CNS morpho-functionality *per se* under physiological conditions: at the neuronal cell level through survival and integrity, through glial regulation, at the transmission efficiency of circuits, grounded on synaptic plasticity, and finally, at the neurocognitive level by regulating long-term memory (LTM). This role of PKR and eIF2α at several organizational and functional levels under physiological conditions points to a potentially relevant role in the early stages of age-related neurodegenerative diseases (Figure 2).

## PKR Role in Age-Related Neurodegenerative Diseases

Based on the extended role described for PKR and eIF2α on CNS morpho-functional levels that we reviewed in the previous section, we identified the potential for this pathway to play a role as a significant regulator of age-related neurodegenerative diseases. We reviewed the possible role of PKR-eIF2α pathway in neurodegenerative diseases in which pathological hallmarks, including progressive neuronal cell death, transmission efficiency defects, and neurocognitive functions decline, can be detected.

### Mechanistic Link Between AD and PKR

The PKR and eIF2α roles over age-related neurodegenerative diseases have been mostly studied for AD in the context of the amyloid cascade hypothesis experimental models. We reviewed the evidence on the role of PKR-eIF2α over AD pathogenic hallmarks. We detailed the studies related to the role of PKR on AD pathogeny in Table 1. A detailed description of the AD models, characteristics, and progression has been extensively detailed elsewhere (De-Paula et al., 2012; Cheng et al., 2018; Bouteiller et al., 2019; Castellani et al., 2019).

**TABLE 1:** Studies involving protein kinase R (PKR) in Alzheimer’s disease.

AD model (*in vitro*/*in vivo*)	PKR active/LOF/GOF	Variable relationship	Neurodegeneration readouts/outcome	References
AD patients	Active	Correlative	PKR accumulation at nuclei in *postmortem* AD brain tissue	Onuki et al. (2004)
5xFAD transgenic mouse	Active	Correlative	p-PKR and p-eIF2α levels are increased in cortical tissue, by immunoblotting	Mouton-Liger et al. (2012a)
Aβ neurotoxicity over primary neuronal culture	PKRi	Functional	PKR pharmacological LOF prevented Aβ_1__–__42_-induced activation of inflammatory pathways, release of TNFα and interleukin (IL)-1β, and inhibited apoptosis	Couturier et al. (2011)
5xFAD transgenic mouse and Aβ_1__–__42_-injected mouse	PKRi	Functional	PKR pharmacological LOF restores deficits in LTM and LTP in both mouse AD models	Hwang et al. (2017)
Aβ toxicity over SH-SY5Y cells and AD patients	PKRi	Functional and correlative	PKR colocalizes with neuronal GSK-3β and tau in AD brains. PKR modulates Aβ induced GSK-3β activation, tau phosphorylation, and apoptosis in neuroblastoma cells	Bose et al. (2011)
AD patients	Active	Correlative	p-PKR in aged brains histology negatively correlates with cognitive scores	Taga et al. (2017)
AD patients	Active	Correlative	A SNP (rs2254958) on the PKR coding gene correlates with AD progression	Bullido et al. (2008)
Thiamine-deficient diet *ad libitum*	PKR-KO mouse or PKRi	Functional	PKR LOF (genetical and pharmacological) reverses Aβ oligomers levels increase in thalamus nuclei, motor deficits, and neurodegeneration induced by thiamine	Mouton-Liger et al. (2015)
Aβ_25__–__35_ neurotoxicity over primary neuronal culture	Active	Correlative	Purified Aβ_25__–__35_ induces PKR phosphorylation	Yu et al. (2007)
Aβ_25__–__35_ neurotoxicity over primary culture of cortical neurons	PKR siRNA	Functional	Purified Aβ_25__–__35_ induces PKR phosphorylation	Lai et al. (2006)
FAD-mutant hAPP mouse and AD patients	Active	Correlative	p-PKR associates with plaques in the FAD-mutant hAPP mouse brain. p-PKR in the hippocampus and the neocortex of AD patients associates with amyloid plaques	Peel and Bredesen (2003)
APPSwe/PS1DE9 mouse and monkeys (*Macaca fascicularis)* exposed to Aβ oligomers	PKRi	Functional	Amyloid-β induces PKR and eIF2α phosphorylation in the brain of mouse and monkeys. Activated PKR correlates with synapse loss and memory impairment	Couturier et al. (2012)
AD patients	Active	Correlative	p-PKR levels at CSF strongly correlates with the severity of cognitive impairment	Dumurgier et al. (2013)
5xFAD mouse	PKR-KO mouse	Functional	PKR LOF (genetic) in the 5xFAD mouse shows reduced BACE1 and Aβ levels, synaptic alterations, neurodegeneration, and neuroinflammation and improves memory defects	Tible et al. (2019)
APPSL/PS1 KI mouse	Active	Correlative	p-PKR and p-eIF2α levels are increased in the cortex of APP_SL_/PS1 KI mouse	Couturier et al. (2010)
Mouse overexpressing the Swedish mutation of 101 amyloid precursor protein (Tg2576)	PKRi	Functional	p-PKR and p-eIF2α levels are increased in the brain of Tg2576 mouse. PKR LOF (pharmacological) alleviates memory deficits in the Tg2576 mouse	Zhang et al. (2016)
Four-month-old ApoE3 and ApoE4 mice	PKRi	Functional	Pharmacological PKR LOF (locally injected) rescues memory impairment and attenuates ATF4 mRNA increased translation in the ApoE4 mouse	Segev et al. (2015)
Aβ_1__–__42_ peptide neurotoxicity over primary neuronal cultures and SH-SY5Y cells	PKR siRNA	Functional	PKR LOF (siRNA) inhibits Aβ_1__–__42_ induced pro-neurodegenerative signaling in nuclei	Morel et al. (2009)
AD patients	Active	Correlative	PKR and eIF2α levels in lymphocytes of AD patients correlates with cognitive and memory test scores	Paccalin et al. (2006)

*AD, Alzheimer’s disease; Aß, amyloid-beta protein; LOF, loss of function; LTM, long-term memory; LTD, long-term depression; TNFα, tumor necrosis factor alpha; IL, interleukin; SNP, single-nucleotide polymorphism; APP, amyloid precursor protein; FAD, familial Alzheimer’s disease; p-PKR, phosphorylated PKR; eIF2α, eukaryotic initiation factor alpha 2; PKRi, PKR pharmacological inhibitor (C16); SH-SY5Y, thrice-cloned subline of bone marrow biopsy-derived line; GSk-3b, glycogen synthase kinase 3 beta; CSF, cerebrospinal fluid; ATF4, activating transcription factor 4; ApoE4, apolipoprotein E4.*

Alzheimer’s disease is characterized by the presence of extracellular senile plaques of the amyloid-beta (Aβ) aggregated protein, intracellular neurofibrillary tangles (NFTs) composed of hyperphosphorylated tau protein, and neuroinflammation (De-Paula et al., 2012; Cheng et al., 2018; Bouteiller et al., 2019; Castellani et al., 2019; Diaz-Zuniga et al., 2020). Senile plaques are composed of Aβ peptides generated after amyloid precursor protein (APP) proteolysis through the amyloidogenic pathway (Chow et al., 2010). Shortly, β-site APP cleaving enzyme 1 (BACE1) cleaves APP, shedding its ectodomain and leaving in the membrane a fragment of 99 amino acids (C99) (Vassar et al., 1999; Bennett et al., 2000). Then, a γ-secretase complex cleaves C99, generating variants of Aβ peptides, being the peptide of 42 residues (Aβ_1__–__42_), the major component of the amyloid plaques and the one with toxic properties (Barrow and Zagorski, 1991; Miller et al., 1993; Klein et al., 1999; Vassar et al., 1999; Bennett et al., 2000; Cai et al., 2001; Luo et al., 2001). Several studies have shown that BACE1 levels and its proteolytic activity are increased in postmortem AD brain samples (Fukumoto et al., 2002; Holsinger et al., 2002; Zhao et al., 2007). According to the amyloid hypothesis (Hardy and Selkoe, 2002), the accumulation and aggregation of Aβ is the triggering event leading to neurodegeneration in AD. These antecedents suggest that elevated BACE1 levels could participate in AD onset or progression. Altogether, these pathological characteristics participate in the neurotoxic mechanism that ultimately leads to a progressive decline of memory function and other cognitive skills (De-Paula et al., 2012; Bouteiller et al., 2019; Castellani et al., 2019).

Protein kinase R can control the levels of BACE1 protein in human neuroblastoma cells exposed to oxidative stress (Mouton-Liger et al., 2012a,b; Taga et al., 2017). The same group has reported that phosphorylated (activated) PKR (p-PKR), p-eIF2α, and BACE1 levels are increased in the AD brain. Moreover, a significant correlation between BACE1 with phosphorylated eIF2α was found (Mouton-Liger et al., 2012b; Taga et al., 2017). These antecedents suggest that PKR-eIF2α could modulate Aβ production. However, little is known about the role of PKR over the specific mechanisms associated with the amyloid hypothesis of AD.

Protein kinase R has also been involved in the mechanism of tau protein phosphorylation. An analysis performed on AD brains and transgenic mouse models found that the distribution of p-PKR matched the distribution of abnormally phosphorylated tau in adjacent sections (Peel and Bredesen, 2003). It has been established that cell lines with reduced PKR expression through RNA interference (RNAi) strategies significantly reduce tau phosphorylation at the 12E8 epitope (serine 262/serine 356), a disease-related phosphorylation site (Azorsa et al., 2010). Notably, a recent work has reported that PKR overexpression and knockdown increase and decrease tau protein and mRNA levels in cell lines, respectively (Reimer et al., 2021). Moreover, the same study showed that PKR directly phosphorylates multiple abnormal and disease-related residues within tau protein (Reimer et al., 2021). Furthermore, this PKR-mediated phosphorylation induces tau displacement from microtubules, promoting a pathological role for tau. Based on this, several authors have proposed that PKR activation links Aβ and tau mechanisms of neurodegeneration (Bose et al., 2011; Amin et al., 2015; Reimer et al., 2021).

Protein kinase R is overexpressed in the brain of patients with AD (Chang et al., 2002; Peel and Bredesen, 2003; Onuki et al., 2004). Activated PKR has been found in neuron cytoplasm, in granule-vacuolar degeneration sites, neuronal nuclei, and around senile plaques by immunohistological analysis of brains derived from AD mouse models and human AD patients postmortem biopsies (Peel et al., 2001; Chang et al., 2002; Peel and Bredesen, 2003; Hugon et al., 2017). In these studies, AD cases showed prominent granular p-PKR immunoreactivity in association with neuritic plaques and pyramidal neurons in the hippocampus and neocortex compared to samples from subjects without dementia (Peel et al., 2001). Interestingly, p-PKR immunoreactivity has also been found distributed within and around the periphery of senile plaques of the Aβ-aggregated protein (Peel et al., 2001; Chang et al., 2002; Peel and Bredesen, 2003; Hugon et al., 2017). Whether this PKR activation patterns and effect over Aβ production are an early event in the disease process or a late consequence of neurodegeneration has not been established. On the other hand, authors have suggested that mutations in the PKR gene are related to an early onset of AD in human patients (Bullido et al., 2008). A 5′ UTR SNP (rs2254958) of the EIF2AK2 (PKR coding gene) has been associated with susceptibility for developing AD at an early age (Bullido et al., 2008). More specifically, this polymorphism is commonly found in AD patients. Compared to other genotypes, the homozygotes of rs2254958 showed earlier (around 3.3 years) onset of AD (Bullido et al., 2008). Consequently, PKR aberrant expression may predispose to AD progression (Peel and Bredesen, 2003). Interestingly, reports from *in vitro* models of AD suggest that PKR is in turn activated by the Aβ peptide (Peel et al., 2001; Hugon et al., 2017).

An interesting report showed that p-PKR is significantly increased in cerebrospinal fluid (CSF) of AD patients when compared with sex-paired and age-matched patients without dementia (Dumurgier et al., 2013). Moreover, when p-PKR was cross-sectionally associated with a standardized cognitive test, the Mini-Mental State Exam (MMSE), it was found that higher levels of p-PKR over the follow-up were correlated with cognitive deterioration (Dumurgier et al., 2013). Interestingly, while CSF Aβ_1__–__42_ levels and p-Tau 181/Tau ratio were also cross-sectionally associated with the MMSE score at the diagnosis, only p-PKR was determined as a biomarker of cognitive decline during the progression of AD. Based on this, authors have proposed that a higher level of CSF p-PKR can predict a faster rate of cognitive decline at the time of AD diagnosis (Dumurgier et al., 2013). This suggests that CNS cellular components actively extrude activated PKR to the extracellular milieu in the context of a progressive worsening of AD.

The role of endogenous activators of PKR in the context of AD has been poorly explored. However, Paquet et al. (2012) reported that PACT and p-PKR have significantly higher colocalization on AD patients’ brains postmortem in comparison to age-matched controls. Furthermore, levels of activated PKR (normalized p-PKR levels) strongly correlate with PACT protein levels on the same samples (Paquet et al., 2012). Interestingly, Aβ_1__–__42_ peptides induce a timely coordinated significant increase in both PKR activation levels and PACT protein levels *in vitro* on the SH-SY5Y human neuroblastoma cell line (Paquet et al., 2012). These data suggest that PACT and PKR could participate in a common cellular response to AD-related neurotoxicity. However, further research is needed to characterize a specific mechanism.

On the other hand, authors have proposed a potential role for dsRNAs, well-described PKR activators, in the context of AD progression. In brief, transcripts with repetitive elements can unspecifically form dsRNA on the cytoplasm (Scheckel et al., 2016). Interestingly, changes in chromatin and epigenetic modifications associated with age-related neurodegenerative diseases promote the derepression of repetitive element transcription due to changes in heterochromatin (Scheckel et al., 2016). Based on this, it has been suggested that this derepression may lead to an increased accumulation of intracellular dsRNA. Notably, RNA-seq-based analysis of global transcriptomes from AD patients versus age-matched controls shows increased levels of transcripts from multiple classes of repetitive elements (Saldi et al., 2019). Furthermore, adenosine-to-inosine RNA editing, a posttranscriptional marker of dsRNA, is also comparatively increased on the same AD patients’ global transcriptomes (Saldi et al., 2019). Thus, a potential role for dsRNAs on the onset or progression of AD has been proposed. However, cellular mechanistic links between dsRNA, PKR, and AD pathogenic markers are currently unknown. In Figure 1, possible stimuli participating in PKR activation are shown on AD context and other age-related neurodegenerative conditions.

As mentioned, it has been widely reported that the activation of the PKR-eIF2α branch of the ISR leads to apoptosis on several cell types, including neurons (Lee and Esteban, 1994; Der et al., 1997; Gil and Esteban, 2000; Scheuner et al., 2006). This proapoptotic role of PKR-eIF2α has been reported to be executed mostly through the canonical apoptotic pathway (Lee and Esteban, 1994; Der et al., 1997; Gil and Esteban, 2000; Scheuner et al., 2006). It has also been shown that PKR inhibition suppresses apoptosis execution in neural cells. Specifically, overexpression of a negative dominant form of PKR (K296R) on neuroblastoma cells inhibits pharmacological ER stress (induced with tunicamycin) and the induction of the apoptotic markers caspase-3 and C/EBP homologous protein (CHOP, also known as GADD153). This apoptosis inhibition correlates with a delay in eIF2α phosphorylation and ATF4 expression (Vaughn et al., 2014). In addition, the role of PKR activation in neuronal survival has been explored on the paradigm of ethanol-induced apoptosis (Qi et al., 2014; Li et al., 2015). This model has shown that ethanol exposure causes neuronal apoptosis in mice’s developing cerebellum (Olney et al., 2002; Qi et al., 2014). There, PKR pharmacological inhibition preserves cell survival under ethanol toxicity in cultured cerebellar granule neurons (Ke et al., 2009). Furthermore, when PKR activity dependence on its endogenous activator RAX is absent through a genetical deletion of the domain of interaction between endogenous activator RAX and PKR (the deficient RAX-binding domain in PKR mouse), Purkinje and granule neurons densities are significantly preserved in response to ethanol when compared to WT mice (Li et al., 2015). Altogether, these results suggest that PKR dynamics regulate neuronal survival in response to neurotoxicity by modulating apoptotic cell death.

To our interest, several reports suggest that the PKR-eIF2α signaling pathway also modulates neuronal apoptosis on AD models. For instance, it has been found that pharmacological or genetic inhibition of PKR significantly reduces Aβ-induced apoptosis (Page et al., 2006). Initially, it was reported that in an AD *in vitro* model based on Aβ deposition, increased tau phosphorylation and neuronal death induced by Okadaic Acid correlates with PKR and eIF2α activation (Kim et al., 2010). Other authors have also reported that cortical neurons from PKR-KO mice exhibit significantly lower apoptotic cell death levels in response to Aβ (Gourmaud et al., 2016). This protective effect of PKR genetic LOF was correlated with significantly lower levels of apoptosis executors, including cleaved poly ADP-ribose polymerase (PARP) and cleaved caspase 3. Levels of Fas-associated protein with death domain (FADD), an adaptor that bridges death receptor signaling to the caspase cascade indispensable for the induction of extrinsic apoptotic cell death, are significantly increased on the cortex of a mouse model of Alzheimer’s disease (5xFAD) at a presymptomatic stage when compared to WT littermates *in vivo* (Couturier et al., 2010). Furthermore, co-immunoprecipitation assays showed that PKR and FADD physically interact in cortex extracts derived from 5xFAD mice, and no interaction is detectable at WT littermates (Couturier et al., 2010). Notably, the authors have determined that Aβ_1__–__42_ induces p-PKR phosphorylation, increases FADD levels, and promotes physical interaction between PKR and FADD in the nucleus of neuroblastoma cells. Even more, PKR gene silencing (RNAi) or treatment with the specific PKR inhibitor, C16, significantly inhibits PKR activation in neuroblastoma cells and inhibits downstream activities of caspase-3 and caspase-8. Taken together, these antecedents suggest that PKR activation promotes neuronal apoptotic cell death in the context of Aβ neurotoxicity in models of Alzheimer’s disease.

Another major characteristic of AD pathogenic hallmark is the glia-mediated neuroinflammation. Briefly, microglial cells execute the innate immunity in the CNS and participate in regulating synaptic plasticity and neuronal circuits activity (Hasan and Singh, 2019; Ikegami et al., 2019; Konishi et al., 2019). Notably, microglia and astrocytes react to pathological stressors by producing and releasing inflammatory mediators that aim to resolve the pathological state. Age-related neurodegenerative diseases operate as chronic pathological stressors over glial cells, which promotes a phenotypical change (glial activation) characterized by a significant increase in the release of inflammatory mediators from glia (Pawate et al., 2004; Lassmann, 2020). The role of glial cells has been largely described for neurodegenerative diseases, including HD (Hsiao and Chern, 2010) and PD (Przedborski and Goldman, 2004). However, a direct contribution of PKR in glial cells has not been explored in HD or PD.

Histological studies of brains from AD patients and AD animal models show a strong colocalization of reactive glial cells with senile plaques and neurofibrillary tangles (Parachikova et al., 2007; Hickman et al., 2008; Lopez-Gonzalez et al., 2015). The inflammatory cascade mechanism during AD associated with Aβ toxicity has been largely reviewed before (Bruni et al., 2020; Kim et al., 2020; Merlo et al., 2020; Webers et al., 2020). In brief, microglia and astrocyte activation participates in Aβ clearance on AD progression’s earlier steps (Ries and Sastre, 2016). However, further AD progression is characterized by an increase in microglial activation. This increase in inflammatory signaling in the latter stages of AD correlates with a significant decrease in Aβ clearance by microglia (Lee and Landreth, 2010; Solito and Sastre, 2012). In turn, Aβ peptide triggers microglial cell activation and induces the release of proinflammatory cytokines (Khandelwal et al., 2011). Specifically, microglia overexpress proinflammatory cytokines such as interleukin (IL)-1β, IL-6, and TNFα, and this promotes neurodegeneration in the later stages of AD (Yamamoto et al., 2007; Di Bona et al., 2008; Forlenza et al., 2009; Wang et al., 2015). Brain inflammation has been reported on several histological analysis of postmortem AD samples (McGeer and McGeer, 2010; Zotova et al., 2010). Astrocytic and microglial cell reactions are often detected surrounding senile plaques. Based on these observations, it has been suggested that glial inflammation may increase synaptic integrity loss and neuronal degeneration during AD.

Notably, the increase in glial proinflammatory cytokines has also been correlated with a significant decrease in hippocampal LTP on several models of AD (Hickman et al., 2008; Ojala et al., 2008; Chakrabarty et al., 2010; Park and Bowers, 2010; Kitazawa et al., 2011; Spooren et al., 2011; Zhao et al., 2011; Sutinen et al., 2012). Thus, inflammatory signaling may be directly related to neurocognitive dysfunction that characterizes AD. Interestingly, proinflammatory cytokines have also been involved in the improvement of neurocognitive functions. Specifically, it has been reported that lipopolysaccharides (LPS) infusion into the insular rat cortex enhances associative taste learning through the increase in glutamatergic AMPA receptors expression and trafficking at synapses (Delpech et al., 2015). Altogether, the relationship between inflammation, transmission efficiency, and neurocognitive functions is highly complex and beyond this review’s scope.

Local and systemic administration of a PKR activator, the dsRNA analogous polyinosinic:polycytidylic acid (poly:IC), has been extensively used as a neuroinflammation model (White et al., 2016). Under this treatment, poly:IC activates inflammatory antiviral responses on neurons and glial cells via Toll-like receptors signaling (Carpentier et al., 2008; Botos et al., 2009; Trudler et al., 2010; Chen et al., 2019). Importantly, it has been found that repeated consecutive peripheral poly:IC injections during 7 days induce a sustained significant increase in hippocampal Aβ levels on mice up to 21 days after last administration (White et al., 2016). Even more, the Aβ increase induced by poly:IC injections strongly correlates with significantly lower performance on the contextual memory test (freezing test) (White et al., 2016). However, a functional relationship between neurotoxicity of Aβ oligomers induced by poly:IC and PKR-eIF2α ISR branch remains unexplored.

Interestingly, authors have proposed that the components of PKR-eIF2α branch participate in CNS response to Aβ-oligomers associated neuroinflammation. Specifically, blocking the TNFα function through a TNFα neutralizing monoclonal antibody (Infliximab) significantly inhibits PKR activation (normalized p-PKR) and eIF2α phosphorylation triggered by Aβ oligomers in neuronal cultures (Lourenco et al., 2013). Notably, TNFα receptor 1 (TNFR1) genetic LOF significantly inhibits hippocampal phosphorylation of PKR and eIF2α in response to local intracerebroventricular (i.c.v.) injection of Aβ_1–42_ oligomers in comparison to WT mice (Lourenco et al., 2013). Concomitantly, TNFR1 genetic LOF completely prevents Aβ-oligomer-induced synapse degeneration on hippocampal neurons *in vitro*. These results suggest that Aβ-dependent activation of TNFα receptors lies upstream of PKR and p-eIF2α *in vivo*. Based on this, authors have theorized that TNFα receptors and the activation of PKR-eIF2α induced by TNFα signaling could participate in memory impairment in response to Aβ oligomers (Lourenco et al., 2013). However, further research is needed to establish a functional link and determine whether this is a neuronal-specific or neuro-glial mechanism. On the other hand, PKR downregulation prevents hippocampal LPS-induced microglial activation and cytokines production (Khandelwal et al., 2011). Specifically, PKR genetic LOF significantly inhibits microglial activation, detected by levels of ionized calcium-binding adaptor molecule 1 (Iba1) and astrocytosis, detected by glial fibrillary acidic protein (GFAP) on the hippocampus of LPS-injected mice in comparison to WT mice (Khandelwal et al., 2011). Concomitantly, PKR genetic LOF also significantly inhibits the increase in brain TNFα and IL-6 induced by LPS (Khandelwal et al., 2011). Thus, PKR participates in a positive feedback between inflammatory signaling related to pathology on the brain. It has been previously reported that LPS i.p. injections promote a significant increase in Aβ peptide on mice brain (Lee et al., 2008). Notably, PKR genetic LOF significantly inhibits LPS-induced Aβ, and BACE1 hippocampal increases protein levels compared to WT mice under the same treatment (Khandelwal et al., 2011). Altogether, these results show that the PKR-eIF2α branch participates in AD-related neuroinflammation as a mediator of Aβ neurotoxicity, where PKR activation is promoted by inflammatory signaling and also promotes an increase in cytokines. Thus, these results suggest that the PKR-eIF2α branch could be a core regulator axis of inflammatory signaling on the memory-related regions of the brain.

Synapse degeneration is another relevant step in AD’s onset and progression (Briggs et al., 2017). Interestingly, synaptic dysfunction occurs first, at presymptomatic stages of AD (Briggs et al., 2017), presumably because of the presence of soluble oligomeric assemblies of Aβ protein (Clare et al., 2010; Robinson et al., 2014; Forner et al., 2017). Furthermore, cognitive dysfunction during AD is strongly correlated with synaptic loss (Forner et al., 2017). Specifically, the morphometric assessment of synapses number in AD demonstrates that synapse loss is the major indicator that correlates with cognitive impairment in patients (Robinson et al., 2014). It has been possible to detect early deficits in synaptic function and plasticity in mouse models of Alzheimer’s disease (Wishart et al., 2006; Hanus and Schuman, 2013). This early synaptic function alteration suggests that structural and functional modifications at synapses may be responsible for the early cognitive decline observed in human patients (Wishart et al., 2006; Hanus and Schuman, 2013). Synapse degeneration and dysfunction are also key pathological events in other dementias and may contribute to the cognitive decline observed during aging and aging-related neuropathologies (Cohen et al., 2013; Namjoshi and Raab-Graham, 2017). However, the role of the PKR-eIF2α branch over synaptic integrity during AD progression remains unknown. A growing body of evidence has proposed that PKR activity inhibition significantly reduces transmission efficiency defects and neurocognitive dysfunction in the context of murine models of AD *in vivo*. For example, pharmacological inhibition of PKR by i.p. injection of C16 in the context of AD model ApoE4 mouse significantly improves the long-term contextual memory compared with ApoE4 vehicle-treated mice (Segev et al., 2015). It is worth mentioning that this pharmacological inhibition of PKR was induced as a pretreatment before memory consolidation training (Segev et al., 2015). In addition, the role of PKR genetic LOF has been functionally assayed over Aβ-oligomer-induced cognitive dysfunction. Specifically, authors have found that i.c.v. injection of Aβ oligomers induces a significant decrease in freezing events on the contextual fear conditioning test in WT mouse (Lourenco et al., 2013). Notably, PKR genetic LOF completely inhibits the decrease in freezing events induced by local Aβ neurotoxicity (Lourenco et al., 2013). Altogether, these results suggest that PKR activation participates in long-term contextual memory impairment induced by the AD pathogenic hallmark mediators.

The role of PKR and eIF2α in transmission efficiency and neurocognitive functions in age-related neurodegenerative diseases has been mostly explored in the 5xFAD AD mouse model. In brief, these 5XFAD mice co-overexpress human APP and presenilin 1 (PS1) carrying five familiar Alzheimer’s disease-related mutations (FAD mutations) in APP and PS1 transgenes (the Swedish mutation: K670N, M671L; the Florida mutation: I716V; the London mutation: V717I) and PS1 (M146L; L286V) driven by the Thy-1 promoter (Oakley et al., 2006). There, it has been described that the Swedish mutation increases Aβ production, and the other mutations contribute to increasing the production of Aβ_1__–__42_. Consequently, in this mouse, five FAD mutations act together to additively increase levels of cerebral Aβ_1__–__42_ neurotoxic peptides (Oakley et al., 2006; Ohno et al., 2006, 2007). Thus, the 5XFAD mouse model develops amyloid deposits (senile plaques) around 2 months of age, consistent with their accelerated Aβ_1__–__42_ production compared to other AD transgenic mouse models (Eriksen and Janus, 2007). At 2 months of age, amyloid deposition begins and accumulates in the hippocampus’ subiculum and specific cortex layers in this mouse model. Then, a sustained increase in Aβ_1__–__42_ deposits fills the hippocampus and cortex of the brain of the 5XFAD mouse up to 6 months of age (Oakley et al., 2006). Concomitantly, 5xFAD mice develop comparatively earlier onset and more aggressive symptoms within amyloid mouse models (Oakley et al., 2006; Ohno et al., 2006, 2007). Related to the participation of PKR in this mouse model, the authors have assayed the effect of pharmacological inhibition of PKR or genetic deletion of PKR over hippocampal LTP and LTD (Lourenco et al., 2013; Zhang et al., 2016; Hwang et al., 2017; Tible et al., 2019). In addition, LTM has been assayed by several authors in this model under the same conditions. It is worth mentioning that authors have described a lack of NFTs in 5xFAD histological samples (Sasaguri et al., 2017). Then, conclusions about the role of PKR and eIF2α obtained on this model under pharmacological or genetic modulations are giving clues mainly about Aβ neurotoxicity as AD pathogenic hallmark. Notably, it has been established that PKR pharmacological and genetic LOF significantly modulates transmission defects and neurocognitive dysfunction during the pathological progression on 5xFAD mouse (Hwang et al., 2017; Tible et al., 2019). There, authors have recently found that genetic PKR LOF in the context of 5xFAD background achieved by double mutation significantly improves LTP on hippocampal slices compared to 5xFAD mouse (Tible et al., 2019). Milder PKR LOF by pharmacological inhibition also significantly improves transmission efficiency on the 5xFAD mice (Hwang et al., 2017). Interestingly, PKR genetic LOFs’ protective effect over synaptic transmission efficiency strongly correlates with memory function maintenance during presymptomatic and symptomatic AD stages in the 5xFAD mouse (Hwang et al., 2017; Tible et al., 2019).

Altogether, the evidence mentioned above reveals an extended role of PKR in AD pathology, where this kinase participates in the demise of neuronal integrity and dysfunctional synaptic transmission efficiency that leads to neurocognitive impairment. This extended role points to interesting pathogenic mechanisms that could be modulated beyond the context of AD.

### The Possible Role of PKR in Huntington’s Disease

Huntington’s disease is a progressive, debilitating, and fatal neurological disorder. Its main symptoms include involuntary or uncontrollable dance-like movements (chorea movements), cognitive and memory impairment, and other psychiatric changes (Ha and Fung, 2012). HD is inherited in an autosomal dominant manner (Cepeda et al., 2007). The mutated gene contains an expansion in the number of CAG repeats in the huntingtin gene (*HTT*) on chromosome 4 (Walker, 2007). HD is typically a late-onset disease, although juvenile variants occur (Cepeda et al., 2007).

Neuropathological changes in HD are characterized by a prominent loss and atrophy of medium spiny projection neurons (MSNs) in the striatum (caudate and putamen) (Vonsattel and DiFiglia, 1998; Gutekunst et al., 2002; Rubinsztein, 2003). Besides, abnormalities in neurons in the cerebral cortex (Vonsattel et al., 1985), substantia nigra, and thalamus, which input to striatal projection neurons (SPN), have been described (Reiner and Deng, 2018). The output tracts to the globus pallidus and substantia nigra from SPN also show abnormalities (Reiner and Deng, 2018). Neuronal cell loss is also evident in other brain regions, including the hippocampus (Vonsattel et al., 1985; Spargo et al., 1993; Utal et al., 1998). The striatal neuron loss is non-prominent in premanifest HD (Vonsattel et al., 1985; Albin et al., 1991; Vonsattel and DiFiglia, 1998), and the early symptoms seem to be driven by striatal neuron circuit connectivity loss and dysfunction.

The striatum, which is highly affected in the disease, is the basal ganglia’s main input nucleus and transforms thalamic and cortical inputs into two output streams, called the direct and the indirect pathways. Although these two pathways’ actions are complex, the basal ganglia circuit’s simplified model proposes that the direct pathway facilitates directed movements, and the indirect pathway terminates or suppresses movements (Smith et al., 1998). Imbalances in the two pathways’ activity are hypothesized to underlie numerous movement disorders, including the ones observed in HD (Galvan and Wichmann, 2007). Medium spiny projection neurons (MSNs) of the striatum, which are GABAergic inhibitory neurons (Lighthall and Kitai, 1983), determine the direct and indirect pathways’ activation.

Another pathological landmark of HD is the presence of aggregated forms of mutant huntingtin protein (mHtt) in neurons. These aggregates include intranuclear and cytoplasmic inclusions, as well as microaggregates. The contribution of these inclusions in neuronal loss observed in HD has not been completely elucidated, but essential processes affected in the disease have been identified (Walker, 2007). The expansion CAG repeats can be bidirectionally transcribed (Cho et al., 2005; Moseley et al., 2006), and several of these mutated genes have been shown to produce aberrant proteins, which are synthesized from multiple reading frames in the absence of AUG in a process called repeat-associated non-AUG (RAN) translation (Zu et al., 2011). One of the disease processes is related to the chronic production of misfolded mHtt, which overwhelms the proteostatic machinery (chaperons, proteasome, and autophagy), leading to a global collapse of the proteostasis network (Soares et al., 2019). Alterations in cell–cell interactions have also been described, including axonal transport and delivery of trophic factors (Soares et al., 2019).

The discovery of the HD gene in 1993 led to the development of genetic models of the disease, which provided material for studies in the earliest stages of disease pathogenesis and mechanistic studies (Cepeda et al., 2010). We address recent findings concerning PKR involvement in HD and its contribution to synaptic transmission efficiency and synaptic integrity. The activation of PKR has been found in tissues derived from HD patients’ postmortem samples. Increased p-PKR levels were detected in the hippocampal tissue of patients with HD, suggesting an association of PKR activation with extrastriatal degeneration (Bando et al., 2005). This result is particularly interesting because the hippocampus’s role in HD pathology in the last years has gained importance (Harris et al., 2019). HD patients showed significant deficits in hippocampal-dependent spatial cognition. Moreover, a correlation was found between the CAG repetitions and the severity of the symptoms, suggesting that deficits relate to HD’s disease process.

Huntington’s disease mouse models have shown alterations in striatal and cortical synaptic transmission. Specifically, the R6/2 mouse model, which carries a fragment of the HD gene (exon 1) and contains 150 CAG repeats (Mangiarini et al., 1996), exhibits a consistent decrease in the frequency of spontaneous excitatory postsynaptic currents (EPSCs) (Cepeda et al., 2003) in striatal MSNs and an increase in spontaneous inhibitory postsynaptic currents (IPSCs) in MSNs (Cepeda et al., 2004, 2010). The increased inhibition of GABAergic neurons would reduce striatal output along the indirect pathway. This may lead to disinhibition of the external globus pallidus and could explain some HD symptoms. Another study performed an electrophysiological analysis of striatal interneurons in the heterozygous Q175 mouse model of HD that contains human *HTT* allele with the expanded CAG repeat (∼179 repeats) (Holley et al., 2019). They found increased excitability of the fast-spiking interneurons (FSIs) and low-threshold spiking (LTS) (Holley et al., 2019). The increase in excitability of FSIs lead to increase in IPSC of MSNs (Koos and Tepper, 1999); interestingly, FSIs are GABAergic cells that provide inhibitory inputs to spiny neuron and degenerate in HD (Gittis et al., 2010). In contrast, LTS neurons, associated with a modulatory role on excitatory synaptic input, are spared from degeneration (Ferrante et al., 1985). This evidence suggests that the increase in the inhibitory activity of MSNs is a common symptom of HD pathology, and a possible role of PKR in mediating the inhibitory activity of GABAergic inhibition on the cortex and striatal MSNs can be considered.

Presymptomatic Huntington’s disease patients often exhibit cognitive deficits before the onset of typical symptoms (Lawrence et al., 1998; Brandt et al., 2002). Learning and memory are believed to depend on changes in synaptic efficacy in certain key brain regions, including the hippocampus and other regions. LTP and LTD, the most studied form of synaptic plasticity, have been largely described to be altered on several HD models (Lione et al., 1999; Lynch et al., 2007; Simmons et al., 2009; Brooks et al., 2012). Indeed, the R6/2 mice displayed age-related alterations in synaptic plasticity at CA1 and dentate granule cell synapses and impaired spatial cognitive performance in the Morris water maze (Murphy et al., 2000). Supporting the premature occurrence of cognitive impairment in HD, LTP has been shown to be reduced in hippocampal slices from presymptomatic Hdh(Q92) and Hdh(Q111) knock-in mice (Lynch et al., 2007). Indeed, the LTP impairment in an early-onset HD mouse model was related to the reduced ability of excitatory synapses in cortical areas to fully respond under low stimulus rates (Usdin et al., 1999). Mouse HD models expressing full-length human mHTT (YAC46 and YAC72) (Hodgson et al., 1999) showed early electrophysiological abnormalities and LTP impairment before any noticeable behavioral abnormalities and any evidence of neurodegeneration or aggregate formation (Hodgson et al., 1999). Moreover, R6/1 HD mice, carrying 115 CAG repeats (Mangiarini et al., 1996), showed reduced hippocampal LTP (Milnerwood et al., 2006). Defects in memory consolidation and cognitive behavior have also been demonstrated in a transgenic HD monkey, including progressive impairment in motor functions and cognitive decline, recognition memory, and spatial memory (Chan et al., 2014). As mentioned, hippocampus-associated behavioral task is impaired in humans affected by the disease (Harris et al., 2019). Hippocampal cell loss, synaptic plasticity abnormalities, and memory impairment are initial events on HD’s pathology. PKR and eIF2α phosphorylation participates in memory consolidation in a bidirectional manner on CA1 hippocampal slices of eIF2α knock-in mice and recombinant PKR-expressing mice (Costa-Mattioli et al., 2007; Jiang et al., 2010). This information strongly suggests that increased PKR levels in HD brains can be partially involved in the impaired LTP and aberrant synaptic plasticity on the hippocampus through eIF2α phosphorylation. An unsolved question in the role of PKR in physiological or pathological conditions is the stimulus that is activating the kinase. In this context, the binding and activation of PKR to RNAs containing another triplet repeat, the CUG sequence, which is the genetic basis of myotonic dystrophy, have been described (Tian et al., 2000).

Protein kinase R also binds preferentially mutant *huntingtin* RNA transcripts containing CAG repeats (Peel et al., 2001), raising the possibility that, in the HD pathological context, PKR activation described in HD mouse models and postmortem samples could be mediated by the binding of expansion of trinucleotide repeat regions. The RAN translation, a pathological phenomenon described in HD, has recently been described as a process regulated by PKR and its phosphorylation (Zu et al., 2020). Genetic deletion of PKR or the expression of a dominant-negative form of PKR inhibits RAN translation. Moreover, metformin, a drug widely used for treating type 2 diabetes and recently tested in neurodegenerative disorders (Gantois et al., 2019; Martinez et al., 2020), showed an inhibiting effect on RAN translation and PKR activation (Zu et al., 2020). It remains to be elucidated how metformin inhibits PKR and its possible effects in neurodegenerative diseases. We detail studies on the role of PKR on HD pathogeny in Table 2.

**TABLE 2:** Reports of protein kinase R (PKR) and age-related neurodegenerative diseases.

AD model (*in vitro*/*in vivo*)	Tissue	Finding	References
HD patients	Brain tissue from HD patients and htt YAC mice	PKR preferentially binds to mutant huntingtin RNA transcripts. p-PKR immunolocalizes with degenerated areas in HD model	Peel et al. (2001)
HD patients	Hippocampal tissue from HD patients	p-PKR is significantly higher and forming aggregates in the nuclei of the CA1, CA2, and CA3 hippocampal regions	Bando et al. (2005)
PD patients	Hippocampal tissue from PD patients	p-PKR is significantly higher CA2 and CA3 hippocampal regions	Bando et al. (2005)
C57Bl/6 mouse treated with MPTP (parkinsonism)	Striatum, midbrain containing the substantia nigra, hippocampus, frontal cortex samples	PKR levels are increased in the striatum and hippocampal tissue and eIF2α phosphorylation is increase in the striatum in response to MPTP	Deguil et al. (2010)

*YAC, yeast artificial chromosome; p-PKR, phosphorylated PKR; CA, *hippocampal* cornu ammonis; MPTP, 1-methyl-4-phenyl-1,2,3,6-tetrahydropyridine; Ser, serine; OLN, oligodendroglial permanent *cell* line; MSA, multiple system atrophy; SH-SY5Y ASYN, alpha-synuclein overexpressing *SH*-*SY5Y* neurons; OLN-AS7, oligodendroglial cells AS7.*

Overall, the evidence shows a role of PKR in HD, either as a synaptic transmission modulator or controlling translation, which reinforces the potential therapeutic role of this kinase in HD.

### A Link Between PD and PKR

Parkinson’s disease is one of the most common age-related brain disorders. PD is defined primarily as a movement disorder, with the typical symptoms being resting tremor, rigidity, bradykinesia, and postural instability. PD is pathologically characterized by degeneration of nigrostriatal dopaminergic neurons and abnormal aggregates of α-synuclein protein, called Lewy bodies, in the surviving neurons (Kalia and Lang, 2015). The presence of these abnormal aggregates of α-synuclein protein is called Lewy pathology. PD patients also display non-motor symptoms, such as cognitive impairment (Muslimovic et al., 2005), recognition memory deficits (Aarsland et al., 2017), and impaired learning (Foltynie et al., 2004; Muslimovic et al., 2005; Aarsland et al., 2009; Elgh et al., 2009). Remarkably, PD and HD patients showed similarities in long-term memory impairment (Scholz et al., 1988); this indicates that similar mechanisms modulate the memory deficits in both neurodegenerative diseases.

In addition to the classic nigrostriatal α-synuclein misfolding and dopaminergic neuronal loss, several mechanisms contribute to the brain changes described in PD, including synaptic dysfunction and loss, mitochondrial dysfunction, retrograde signaling impairment, and altered neurotransmitter activity, among others (Aarsland et al., 2017). Compared with the motor symptoms, little is known about the mechanisms underlying cognitive decline in PD, and several key questions remain unresolved. The evidence from postmortem studies indicates that Lewy body pathology in limbic and cortical areas is the main pathological hallmark of PD’s cognitive impairment. The model proposed is that α-synuclein pathology spreads from areas in the lower brainstem or olfactory bulb (or extra-CNS territories like the gut or other areas innervated by the vagus nucleus) to the midbrain, forebrain, and limbic structures, and, finally, neocortical regions (Braak et al., 2003; Sauerbier et al., 2016).

The contribution of PKR in PD has been poorly explored. However, an interesting set of evidence suggests that PKR may play a role in PD pathogenesis. Specifically, postmortem biopsies from PD patients show a significant increase in activated PKR in hippocampal neurons compared to age-matched controls. Furthermore, activated PKR was also significantly increased on nuclei from hippocampal lysates from the same patients (Bando et al., 2005). Interestingly, murine models of PD reproduce this significant increase in activated PKR at the hippocampus (Deguil et al., 2010). Specifically, parkinsonism in mice induced by intraperitoneal injection of 1-methyl-4-phenyl-1,2,3,6-tetrahydropyridine (MPTP) correlates with a significant increase in activated PKR at the hippocampus (Deguil et al., 2010). Interestingly, a preclinical treatment against PD significantly reduces activated PKR at the hippocampus and improves spatial memory on the Morris water maze paradigm (Deguil et al., 2010). Furthermore, additional strong evidence can be considered to involve PKR as kinase participating in PD. It has been described that the extent of amyloid plaque pathology observed in PD is a significant contributor to the cognitive decline observed in the disease (Compta et al., 2011; Aarsland et al., 2017), suggesting a possible common mechanism driving cognitive impairment observed in AD and PD. Another common aspect can be found between AD and PD. Synaptic dysfunction followed by synaptic loss is likely to be the early and key events in AD (Terry et al., 1991). Emerging evidence has shown synaptic alterations in PD patients (Matuskey et al., 2020). Using *in vivo* high-resolution positron emission tomographic imaging and postmortem autoradiography derived from patients’ samples, the authors showed decreased levels of synaptic vesicle glycoprotein 2A (SV2A) in PD patients (Matuskey et al., 2020). Notably, most of the genes implicated in PD (e.g., *SNCA*, *LRRK2*, *DJ-1*, *PINK1*, and *PRKN*) have a critical role in synaptic function, and knockout mouse for each of the genes has demonstrated disruption of synaptic plasticity and neurotransmitter function (Plowey and Chu, 2011; Belluzzi et al., 2012; Abeliovich and Gitler, 2016).

Cognitive impairment is also detected in mouse models of the disease, in which partial lesions of dopaminergic and noradrenergic inputs to the striatum and hippocampus are induced with 6-hydroxydopamine. This mouse displayed reduced long-term novel object recognition and decreased LTP, predominantly in the dentate gyrus (Bonito-Oliva et al., 2014). Concomitantly, the application of extracellular α-syn oligomers in rat hippocampal brain slices impairs LTP (Martin et al., 2012). On the other hand, overexpression of α-syn induced impairment in short-term memory and spatial learning in rats principally due to α-syn accumulation primarily in the CA2 region. Thus, processes controlled by PKR are also altered in PD mouse models. We detail studies on the role of PKR on PD pathogeny in Table 2.

The phosphorylation of eIF2α in the substantia nigra has been described in the postmortem tissue from PD cases, and in the same study, the activation (phosphorylation) of another ISR kinase, the ER stress sensor PERK (Hoozemans et al., 2007), was also detected. On the other hand, another study showed strong induction of phosphorylated PKR in hippocampal neurons (Bando et al., 2005), but the phosphorylation of eIF2α was not analyzed. It is possible to consider that PKR could be mediating the phosphorylation of eIF2α in other regions, i.e., substantia nigra, and driving cell death events observed in the disease.

The stimulus activating PKR in PD is still unknown. In this context, an association between viral infections mediated by herpes simplex virus (HSV) and influenza virus A and increased PD incidence has been found (Olsen et al., 2018). It has been proposed that HSV influenza virus A infections may lead to PD pathology. Viral transcripts, possibly detected by PKR, could be part of the mechanism underlying the association proposed.

## Concluding Remarks

The role of PKR seems to be relevant in physiological and pathological conditions but with completely different consequences. PKR could be considered as a regulator of synaptic efficiency transmission and, consequently, a neurocognitive regulator. The identity of the stimulus that activates the kinase in normal conditions remains to be determined. In pathological conditions, PKR seems to be a cell death regulator, resulting in an interesting candidate for therapeutics strategies. The abnormalities observed in aged-related neurodegenerative diseases could have a common regulator, PKR, mediating apoptotic signals, synaptic transmission deficiencies, and neurocognitive dysfunction.

## Author Contributions

NM, FG, and SM contributed equally to the final manuscript. All authors contributed to the article and approved the submitted version.

## Conflict of Interest

The authors declare that the research was conducted in the absence of any commercial or financial relationships that could be construed as a potential conflict of interest.
